# The Association Between Iron Deficiency at Diagnosis, Female Sex, and Tissue Transglutaminase Antibody Normalization in Pediatric Celiac Disease

**DOI:** 10.7759/cureus.62951

**Published:** 2024-06-23

**Authors:** Eyal Zifman, Dana Schujovitzky, Yaara Moskovitz-Hivert, Tut Galai

**Affiliations:** 1 Pediatric Gastroenterology, Meir Medical Center, Kfar Saba, ISR; 2 Pediatrics, Meir Medical Center, Kfar Saba, ISR

**Keywords:** iron deficiency, anemia, tissue transglutaminase, remission, ferritin

## Abstract

Background: Iron deficiency (ID) is one of the most common manifestations of Celiac disease (CD). We aimed to determine whether ID at CD diagnosis affects tissue transglutaminase antibody (TTG) normalization rate among pediatric CD patients adhering to a gluten-free diet (GFD).

Methods: We conducted a retrospective, observational cohort study that enrolled CD subjects aged 2-18y, diagnosed between Jan 2016 and Dec 2020. Demographic and laboratory data were collected at diagnosis and one year after adherence to GFD. ID was determined according to hemoglobin and ferritin levels. We compared CD subjects with and without ID at CD diagnosis in relation to TTG normalization at one year.

Results: Our cohort included 118 pediatric CD subjects. At diagnosis, 61 (51.7%) of CD subjects had ID, of whom 27 (44.3%) were female, compared to 46 (80.7%) females in the non-ID group (p<0.001). Median age at CD diagnosis was 5.7y (IQR 4-8.4, range 2-14) and 7.2y (IQR 4.7-10.8, range 0.9-16), and among those with and without ID, respectively (p=0.1). After one year of adherence to GFD, TTG normalization was achieved in 38 (65.5%) and 28 (53.8%) of those with and without ID at CD diagnosis, respectively (p=0.21). However, TTG normalization was achieved in 38 (79.2%) of males compared to 42 (49.4%) of females (p=0.001).

Conclusions: ID at CD diagnosis was not associated with lower rates of TTG normalization at one year among pediatric patients adhering to GFD. However, TTG normalization at one year was significantly more frequent among male subjects compared to females.

## Introduction

Celiac disease (CD) is a chronic immune-mediated disorder caused by an inflammatory response to gluten ingestion in genetically susceptible individuals. The diagnosis of CD relies upon specific serological tests and characteristic histological changes found in the duodenal biopsies. A "no-biopsy" method to diagnosis has become more prevalent and was endorsed by the European Society of Pediatric Gastroenterology Hepatology and Nutrition (ESPGHAN) guidelines for the diagnosis of Celiac disease in specific patients [1-2]. The main serological test used for the diagnosis of CD is tissue transglutaminase antibodies (TTG), an immunoglobulin A (IgA) based antibody, which was proven to be very sensitive and specific for Celiac disease. The only proven therapy for CD is lifelong adherence to a gluten-free diet (GFD). CD patients' follow-up relies upon TTG measurements, and the resolution of symptoms and assurance of dietary compliance and adequate growth constitute the mainstay of follow-up for these patients. Normalization of the TTG levels is often termed "serologic remission" (SR) for CD patients during follow up.

Iron deficiency (ID) and iron deficiency anemia (IDA) are among the most common extra-intestinal manifestations of celiac disease and are thought to have multifactorial etiology, including hampered intestinal absorption of iron secondary to reduced surface area in the proximal small intestine as well as from reduced expression of important iron regulatory proteins due to the chronic inflammation [3]. Rarely, blood loss from the upper and lower gastrointestinal tract may also contribute to the development of IDA [4-5].

It was previously shown that the decrease in TTG levels might be prolonged and may take more than a year [6,7]. The inadequate decline of TTG levels may point to GFD non-adherence, thus mandating a more stringent follow-up and frequent dietary counseling. Moreover, TTG normalization correlated well with histological remission [8]. Predicting which patient is more likely to normalize their TTG levels later rather than sooner may obviate the need for frequent follow-up visits and unnecessary scrutiny of patients' diets. The current study aimed to determine whether iron deficiency with and without anemia at CD diagnosis is related to the later normalization of TTG levels.

This article was previously posted to the Research Square preprint server on January 23, 2024.

## Materials and methods

We performed a retrospective observational cohort study using data extracted from electronic patient files of CD subjects followed by the pediatric gastroenterology service. The Meir Medical Center's institutional review board approved the study, and the need for informed consent was waived (MMC-20-0021). The trial was registered in Clnicaltrials.gov (NCT05675280).

The diagnosis of CD was made in accordance with the ESPGHAN guidelines, and all subjects aged 2 to 18 years who were diagnosed from January 2016 to December 2020 were enrolled [1]. Subjects not adherent to GFD were excluded, as well as subjects with missing data in which determination of SR could not be determined. We also excluded subjects diagnosed with total IgA deficiency and those having other possible etiologies for anemia (e.g., thalassemia, spherocytosis, inflammatory bowel disease, chronic kidney disease, connective tissue disease).

Adherence to GFD was ascertained by specific questioning as part of the visit, both by a pediatric gastroenterologist and by an experienced dietitian. Anemia was defined as hemoglobin <11g/dL for children ≤5 years old, <11.5g/dL for children 5−11 years old, <12 g/dL for children 12-14 years old and <13g/dL for boys and <12g/dL for girls >15 years old [9]. Iron deficiency was defined as ferritin level <12ng/mL.

The primary outcome was the rate of SR determined by TTG level normalization after 1 year of GFD adherence among subjects with and without ID.

Statistical analysis: Categorical variables are reported as frequency and percentage. Continuous variables were evaluated for normal distribution using histogram, Q-Q Plots, and Shapiro-Wilk test and reported as median and interquartile range (IQR) for non-normally distributed variables or mean and standard deviation (SD) for normally distributed variables. Continuous variables were compared using an independent sample Mann-Whitney, while categorical variables were compared using the chi-square test. Statistical analyses were performed with SPSS-24 software (IBM, Armonk, NY).

## Results

We enrolled 127 pediatric subjects diagnosed with CD between January 2016 and December 2020. We excluded 3 patients who did not adhere to GFD and 3 subjects with missing data. We also excluded 1 subject with concurrent juvenile idiopathic arthritis and 2 subjects with eosinophilic esophagitis (which by themselves may cause anemia of chronic disease or iron deficiency). Our final cohort comprised 118 subjects - 91 (64%) were female, and the median age was 5.9y (IQR 4.4-9.8). Demographic data and laboratory parameters at diagnosis and after one year of adherence to GFD are presented in Table 1. At diagnosis, 61 (51.7%) had ID, and of them, 23 (37.7%) had IDA. In the ID group, 27 (44.3%) were female, compared to 46 (80.37%) among subjects without ID (p<0.001). Median age at CD diagnosis was 5.7y (IQR 4-8.4, range 2-14) among those with ID and 7.2y (IQR 4.7-10.8, range 0.9-16) in those without ID (p=0.1). Median TTG levels at diagnosis were higher in the ID group vs. subjects without ID (16.7 vs. 15.4 times the upper limit of norm (xULN), p=0.023).

**Table 1:** Demographic and laboratory data. The data is presented as median (IQR) or N (%) as applicable * p-value is significant <0.05 ID: Iron deficiency; IQR: Interquartile range; xULN: Times upper limit of norm; TTG: Tissue-transglutaminase antibody

	ID at diagnosis (N=61)	No ID at diagnosis (N=57)	p-value	Entire cohort
Median age at diagnosis, y (IQR)	5.7 (3.95-8.35)	7.2 (4.65-10.75)	0.098	5.9 (4.38-9.8)
Sex, female, n (%)	27 (44.3%)	46 (80.7%)	<0.001>	91 (64.1%)
Median ferritin at diagnosis, ng/mL (IQR)	7.5 (5.72-11.25)	22.6 (15.2-32.8)	<0.001>	13.6 (7.1-22.45)
Median hemoglobin at diagnosis, gr/dL (IQR)	12 (11.2-12.4)	12.6 (12.15-13.1)	<0.001>	12.4 (11.6-12.83)
Anemia at diagnosis, n (%)	23 (37.7%)	5 (8.8%)	< 0.001>	33 (23.2%)
Median TTG at diagnosis, xULN (IQR)	16.7 (11.55-72.45)	15.35 (4.83-34.1)	0.023	16.7 (8.1-51.45)
Median ferritin at 1y, ng/mL (IQR)	14.45 (8.6-21.73)	23.5 (16.2-35.7)	<0.001>	20 (12.35-28.7)
Iron deficiency at 1y, n (%)	23 (48.9%)	6 (13.6%)	<0.001>	61 (42.7%)
Median hemoglobin at 1y, gr/dL (IQR)	12.7 (12.15-13.3)	12.7 (12.2-13.2)	0.932	12.65 (12.2-13.2)
Anemia at 1y, n (%)	8 (13.8%)	5 (9.6%)	0.498	14 (10.5%)
Median TTG at 1y, xULN (IQR)	0.7 (0.3-1.8)	0.9 (0.52-3.00)	0.130	0.8 (0.45-2.2)
Serological remission at 1y, n (%)	38 (65.5%)	28 (53.8%)	0.212	80 (60.2%)

SR was achieved in 38 (65.5%) and 28 (53.8%) of those with and without ID at CD diagnosis, respectively (p=0.212). However, despite this, ID subjects had higher median absolute TTG levels at one year compared to those without ID - 8.7 (IQR 4.1-16.5) vs. 7.2 (IQR 3.1-22), respectively (p=0.037).

Note that, in one year, SR was achieved in 38 (79.2%) of males compared to 42 (49.4%) of females (p=0.001). This finding remained significant on multivariate analysis correcting for age, ferritin, and hemoglobin levels.

In regards to the ferritin levels at one year, median levels were 14.5ng/mL (IQR 8.6-21.8) and 23.5ng/mL (IQR 16.2-35.7) for those with and without ID at diagnosis, respectively (p<0.001).

## Discussion

In this study, we found that iron deficiency at the time of CD diagnosis did not impact the rate of SR manifested by TTG normalization among pediatric patients with CD adhering to GFD. Moreover, IDA at diagnosis was not associated with higher rates of iron deficiency or higher median TTG levels at 1 year after GFD initiation. However, we detected higher SR rates in one year among male subjects than females. Interestingly, 4 (5.1%) subjects who did not have anemia at CD diagnosis were found to have anemia at one year, although none of them had iron deficiency as the cause of anemia.

Our study is in line with a previous study, which reported no significant difference in the serological response between children with and without anemia at diagnosis and after a median follow-up of one year [10]. This study also found that iron-deficient children had higher median TTG values at diagnosis and after one year of GFD adherence than children without iron deficiency. However, the latter finding has no clinical significance since values are within the normal range. 

Several other studies have examined the rate of TTG normalization itself in pediatric CD. Girdewicz et al. found that TTG normalization varied greatly according to baseline TTG and anti-endomysial antibody levels, ranging from 20.3% to 64.6%, with lower rates of TTG normalization in those with initial TTG levels ≥10 ULN [5]. On the other hand, Doyev et al. presented short median periods for TTG normalization of 4.5m (IQR 3-9) [11]. To note, subjects with histologic involvement confined to the duodenal bulb had a milder phenotype and faster median time to TTG normalization of 3m vs. 5m compared to subjects in whom the 2nd part of the duodenum was affected. A large retrospective study by Krauthammer et al. has shown higher TTG normalization rates at one year after diagnosis of 73% in a recent decade (2009-2018) compared to 53% in the preceding one [12].

Recently, Ashton et al. found that normalization of TTG levels occurs within 6-12 months for 50% of the patients in their study [13]. They also found that higher TTG levels at diagnosis take longer to normalize and that the level does not appear to be related to growth problems or symptoms.

Deora et al. looked into micronutrient deficiencies at diagnosis and during follow-up of pediatric CD patients [3]. They reported low ferritin levels in 31 out of 90 (34.4%) children at diagnosis and 17 (20.5%) at 18 months after diagnosis. They did not collect data at 12m after diagnosis. Ziv-Baran et al. found anemia at CD diagnosis correlated with TTG titer but not with histological Marsh classification among 432 pediatric CD patients [14]. In our cohort, iron deficiency at CD diagnosis was present in 57 (54.8%) but only in 25 (24%) after one year of GFD. However, we did not account for Marsh classification scores in our cohort.

Another finding in our study was that there was a higher SR at one year among males compared to females. This finding is in line with a recent multicenter large cohort of children with celiac disease on a GFD from Italy, which reported female sex as a risk factor associated with a longer time to serology normalization. TTG>10 ULN at diagnosis, age 7-12 years old, poor compliance to diet, and non-Caucasian ethnicity were other significant independent risk factors found [15].

Our study has several limitations. First, since this was a retrospective study, only data from the subjects' electronic medical files could be collected. Secondly, TTG testing was performed using more than one kit, so absolute values and different thresholds for normal values had to be accommodated. This is why we presented data as times ULN, obviating absolute TTG levels. Thirdly, being a single-center study, the cohort size was small. It is possible that a larger cohort would have enabled us to better associate ID at CD diagnosis with TTG normalization rates at 1 year.

## Conclusions

To conclude, ID at the time of diagnosis was not associated with lower rates of TTG normalization at one year among pediatric CD patients adhering to GFD. However, TTG normalization was more prevalent among males than females. Future and larger prospective studies are mandated to elucidate better the relation between ID at CD diagnosis and the rate of TTG normalization.
